# The Efficacy of Prehabilitation Programs in Improving the Quality of Life, Anxiety, and Depression of Individuals Undergoing Surgery: A Meta‐Analysis of Randomized Controlled Trials

**DOI:** 10.1002/pchj.70080

**Published:** 2026-02-05

**Authors:** Xi Qu, Yue Guo, Guolong He, Chunjing Zhang, Hong Chen

**Affiliations:** ^1^ Department of Nursing Tongji Hospital, Tongji Medical College, Huazhong University of Science and Technology Wuhan China

**Keywords:** anxiety, depression, meta‐analysis, prehabilitation, quality of life, surgery

## Abstract

To assess the efficacy of prehabilitation programs in improving quality of life and alleviating anxiety and depression among adults undergoing surgery, a systematic review and meta‐analysis was conducted by searching seven major biomedical databases (CNKI, Wanfang, Cochrane Library, PubMed, Web of Science, Embase, and Sinomed) from inception to October 30, 2025. Randomized controlled trials evaluating prehabilitation interventions in surgical patients were included. Eleven studies met inclusion criteria. Prehabilitation significantly improved postoperative quality of life (effect size = 4.68, 95% CI [1.18, 8.18], *p* < 0.05) and reduced depressive symptoms (effect size = −0.13, 95% CI [−0.26, −0.01], *p* < 0.03), whereas its effect on anxiety was not significant (effect size = 0.01, 95% CI [−0.13, 0.14], *p* = 0.92). Subgroup analyses indicated that benefits were most evident in the medium‐term period, while long‐term effects were minimal. No publication bias was observed, and the overall quality of evidence was moderate. Prehabilitation is effective in enhancing quality of life and reducing depression in surgical patients; however, its impact on anxiety remains inconclusive. Optimal effects may be associated with structured, multimodal prehabilitation programs and medium‐term follow‐up. Future randomized trials should examine program components, delivery modes, and long‐term outcomes to refine clinical implementation.

## 1 Introduction

Surgical procedures are a cornerstone of modern medicine. In recent years, the number of surgical procedures has increased significantly due to advancements in technology and techniques that have made surgeries safer and more accessible (Madsen et al. 2023). However, the perioperative period poses substantial challenges for patients, often leading to adverse outcomes if not managed appropriately (Han et al. 2023; Kenny et al. 2022). The transition from preoperative assessment to postoperative recovery can be particularly daunting, as patients frequently experience a range of physical and psychological stressors. Common issues that arise include postoperative pain, anxiety, and depression, which not only degrade the quality of life (QoL) but can also hinder recovery, leading to longer hospital stays, increased morbidity, and reduced overall satisfaction with the surgical experience (Taha et al. 2021; Zhang et al. 2024).

Prehabilitation refers to proactive interventions provided to patients to enhance their preoperative health and well‐being before the stress of surgery (Deprato et al. 2022; Steffens et al. 2018). It encompasses a variety of interventions, typically including structured exercise training, nutritional optimization, and psychological support (e.g., cognitive‐behavioral therapy, relaxation techniques), aimed at improving patients' physical and psychological health before surgery. The primary goal is to enhance postoperative outcomes and QoL by optimizing patients' preoperative condition (Steffens et al. 2018; Timmerman et al. 2011). Several studies have demonstrated that prehabilitation can improve physical functioning and reduce complications after surgery (Gillis et al. 2014, 2018; Li et al. 2013). For instance, a meta‐analysis by Gillis et al. revealed that a prehabilitation program comprising physical exercise and nutritional counseling significantly diminished postoperative complications in patients undergoing colorectal surgery (Gillis et al. 2018). Moreover, growing evidence indicates that prehabilitation may positively influence mental health (Liang et al. 2024; Lotzke et al. 2019; Peng et al. 2023). Depression and anxiety frequently occur in patients undergoing surgery, and these psychological factors can negatively affect recovery. Research by Lotzke et al. (2019) highlighted that psychological interventions, involving cognitive‐behavioral therapy (CBT) integrated into prehabilitation programs, can reduce anxiety and depression levels preoperatively.

Although their increasing prevalence, there exists significant diversity in the design and execution of prehabilitation programs, resulting in variations in outcomes. The study of prehabilitation programs' effectiveness on QoL, anxiety, and depression in individuals undergoing surgery is a timely and significant area of research. Conducting a meta‐analysis of RCTs to address the deficiencies in existing evidence might yield significant insights and facilitate the incorporation of prehabilitation into conventional perioperative care regimens. This has the potential to enhance patient outcomes and decrease healthcare expenses related to surgical complications and extended recovery durations.

## 2 Methods

### 2.1 Study Design

This review complied with the PRISMA 2020 requirements (Page et al. 2021) and the GRADE approach was used to assess the quality of evidence for main outcomes. The review was registered with PROSPERO under registration number CRD42024558842. Ethical approval and informed consent were unnecessary, as all data included in this study were obtained from previously published research.

### 2.2 Search Strategy

We conducted a systematic search across Embase, PubMed, Cochrane Library, Web of Science, CINAHL, and PsycINFO from the establishment of these databases until October 30, 2025. We employed a synthesis of medical subject headings and free‐text terminology pertinent to prehabilitation, surgery, quality of life, depression, and anxiety. Additionally, we conducted a manual review of the reference lists from the included papers and related reviews to uncover additional references.

### 2.3 Study Eligibility Criteria

The criteria for inclusion were as follows: (1) Participants consisted of adult patients (≥ 18 years) undergoing elective surgery; (2) Studies employed prehabilitation interventions (specifically including exercise training, nutritional support, psychological interventions, or a combination); (3) Comparators included routine treatment, standard therapy, normal care, or no intervention; (4) Outcomes assessed included at least one of quality of life, anxiety, or depression; (5) Study design was randomized controlled trials (RCTs) or pilot RCTs; and (6) Languages were restricted to English.

We excluded studies that: (1) were duplicate publications; (2) consisted of conference proceedings, abstracts, research protocols, letters, comments, or editorials; and (3) presented incomplete data with no answers following author contact.

### 2.4 Study Selection and Data Extraction

**FIGURE 1:**
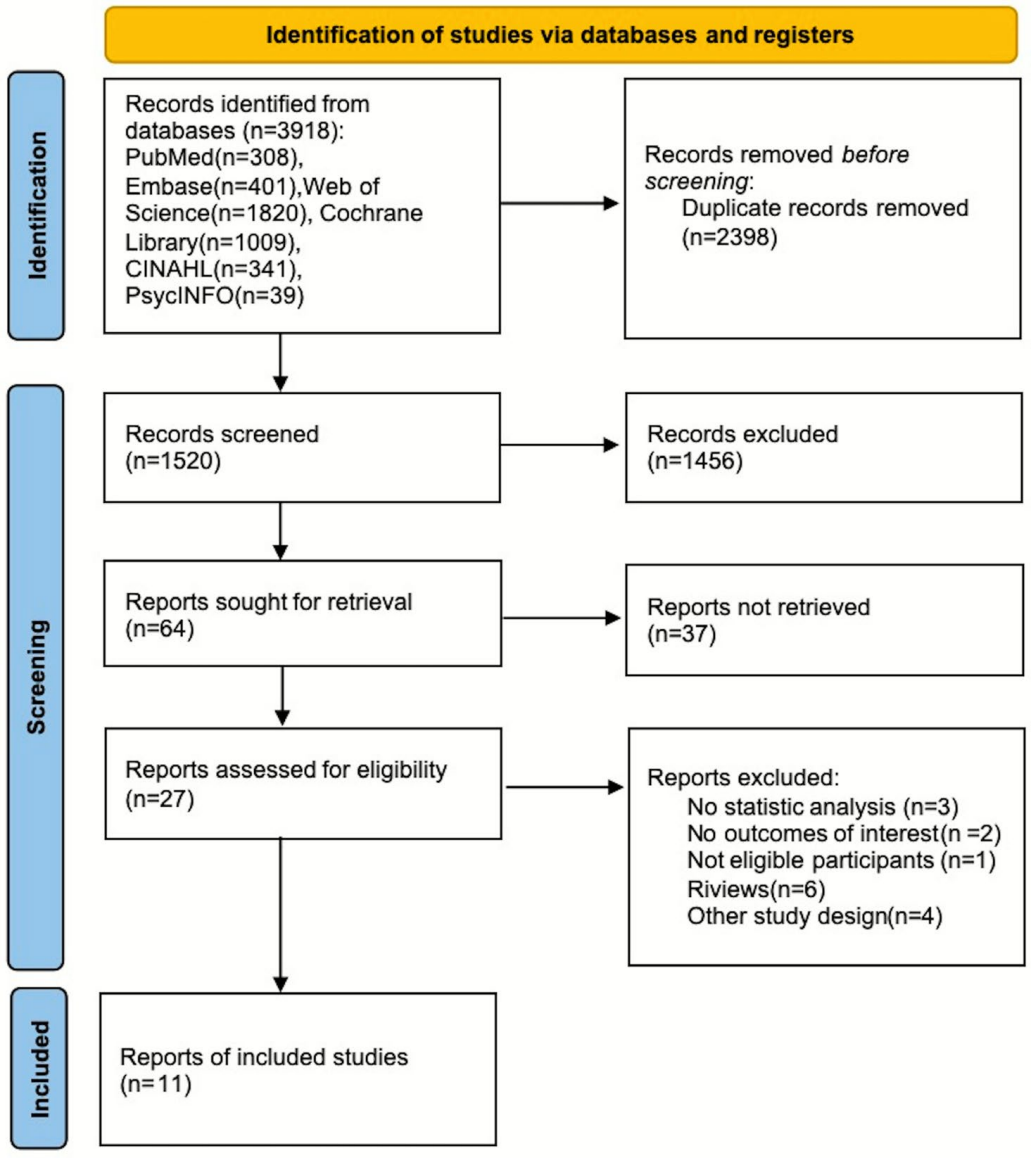
Flowchart of study identification.

All duplicate articles were removed using Endnote X9. Two authors concurrently evaluated the titles and abstracts of articles to ascertain their compliance with the inclusion criteria. Disagreements were addressed through dialog or consultation with a third author. Figure 1 is a flowchart of the screening pipeline and selection procedure.

Data extraction from the included studies was conducted separately by two authors utilizing a pre‐established data extraction form. The extracted data comprised the first author, year of publication, country of study, sample size, age (mean and standard deviation), brief overview and essence of interventions (intervention type, frequency and duration, intensity and targets, and instructor), total number of male and female patients (at pre‐ or post‐intervention), control treatments, outcome indicators, and assessment phases.

### 2.5 Quality Appraisal

Two reviewers independently evaluated the methodological quality and risk of bias of the included studies utilizing the bias risk assessment technique from the Cochrane Handbook for Systematic Reviews of Interventions Version 5.1.0. The Cochrane tool evaluates seven domains for randomized controlled trials: random sequence generation (selection bias), allocation concealment (selection bias), blinding of participants and personnel (performance bias), blinding of outcome assessment (detection bias), incomplete outcome data (attrition bias), selective reporting (reporting bias), and other potential biases. Each category is classified as “low risk,” “high risk,” or “unclear risk” of bias. Disputes were settled by negotiation or consultation with an external reviewer. The GRADE framework was applied to rate the overall quality of evidence for each primary outcome (QoL, anxiety, depression).

### 2.6 Statistical Analysis

We conducted heterogeneity tests and meta‐analysis using Review Manager 5.3 to estimate intervention effects, presenting results as mean differences (MD) and standard deviation (SD) in forest plots. Statistical significance of the overall effect was determined by a two‐tailed *P* value < 0.05. Additionally, we evaluated statistical heterogeneity among included studies utilizing the *I* ^*2*^ statistic and *P* value. Data were aggregated and examined utilizing a fixed‐effects model where *I* ^*2*^ ≤ 50% and *p* > 0.1. In instances of increased heterogeneity, signifying considerable variations among research, we utilized a random‐effects model to get more cautious estimates. The robustness and reliability of the aggregated results were assessed through a sensitivity analysis that utilized a one‐study‐out procedure. A funnel plot was employed to investigate potential publication bias. Begg's funnel plot and Egger's test were implemented to evaluate publication bias in instances of suspected bias (STATA 12.0: *p* < 0.1 was considered statistically significant). A subgroup analysis was performed based on surgical type to explore potential sources of heterogeneity.

## 3 Results

### 3.1 Study Characteristics

This review encompassed 11 randomized controlled trials. Three studies were performed in Canada (Baillot et al. 2018; Brahmbhatt et al. 2024; Coca‐Martinez et al. 2023), three studies in the United Kingdom (Akowuah et al. 2023; Allen et al. 2022; Furze et al. 2009), and one study each in Portugal (Machado et al. 2024), the Netherlands (Berkvens et al. 2021), the United States (Brown et al. 2012), Italy (Ferrara et al. 2008), and Sweden (Heiman et al. 2022). The research encompassed operations involving surgery of the digestive system (*n* = 2) (Baillot et al. 2018; Berkvens et al. 2021), breast cancer surgery (*n* = 2) (Brahmbhatt et al. 2024; Heiman et al. 2022), arthroplasty surgery (*n* = 2) (Brown et al. 2012; Ferrara et al. 2008), lung surgery (*n* = 1) (Machado et al. 2024), endovascular revascularization (*n* = 1) (Coca‐Martinez et al. 2023), cardiac surgery (*n* = 2) (Akowuah et al. 2023; Furze et al. 2009), and esophageal surgery (*n* = 1) (Allen et al. 2022). The features of the investigation are summarized in Table 1.

**TABLE 1:** Characteristics of included studies.

Study/country	Participants (EG/CG)		Surgery type	Intervention		Measure points	Outcomes/measures
	Sample size	Mean age		EG	CG		
(Machado et al. 2024), Portugal	20/21	68.1 ± 9.3	Confirmed or suspected lung malignancy	PHET, a home‐based exercise regimen following oncology guidelines, combines aerobic and resistance training. Aerobic sessions are thrice weekly, starting at 30 min and increasing to 40 min post‐second week. Resistance training occurs twice weekly with six exercises, initially two sets of 15 reps per exercise, progressing to three sets post‐second week. Weekly telephone supervision by a therapist is included	Standard preoperative treatment involved weekly telephone consultations with routine inquiries on tiredness, discomfort, and dyspnea symptoms, excluding any formal exercise training	Baseline Before surgery 1 month after surgery	HRQoL (QLQ‐C30)
(Akowuah et al. 2023), UK	91/89	/	Cardiac surgery	The intervention group received standard care plus cardiac prehabilitation, including initial fitness assessment, supervised 2 × 60‐min/week. Exercise for 4 weeks, home program up to 45 min/day, and twice‐daily inspiratory muscle training using an inspirometer until surgery day	The control group received standard pre‐operative care, including routine consultations with clinical staff	Baseline Pre‐operative 6 weeks after surgery 12 weeks after surgery	EQ‐5D
(Allen et al. 2022), UK	26/28	64.0 ± 8.0	Esophageal surgery	Participants engaged in resistance workouts (2 sets of 12 repetitions at RPE 12–14) targeting key muscle groups with free weights or bands, got customized dietary treatments to enhance calorie and protein consumption, and attended six sessions of psychological support via Medical Coaching	Usual care	Baseline 2 weeks after surgery 6 weeks after surgery 6 months after surgery	EORTC QLQ‐C30 Becks' anxiety inventory Becks' depression inventory
(Baillot et al. 2018), Canada	13/12	44.5 ± 8.8 41.1 ± 10.3	Bariatric surgery	Patients had normal treatment with the PreSET program, which included three weekly 80‐min sessions of endurance and strength training, supplemented by monthly aquagym sessions	Participants received standard care, comprising personalized lifestyle counseling and biweekly supervised “Motivated Club” meetings for education and motivation	1 year after surgery	Weight‐related quality of life (WRQOL)
(Berkvens et al. 2021), Netherlands	11/11	/	Abdominal wall reconstruction	The intervention (preoperative exercise therapy (PexT)) was a three‐month‐long exercise program that included cardiovascular, strength, and respiratory muscle training, and was conducted under the direct supervision of a physiotherapist	Usual care	Baseline After 3 months' intervention	SF–36
(Brahmbhatt et al. 2024), Canada	35/37	57.4 ± 11.94 54 ± 10.69	Breast cancer	Participants in the intervention got a multimodal prehabilitation program that included a personalized exercise regimen, nutritional assistance, and stress management counseling during neoadjuvant chemotherapy	Usual care	Baseline after NACT completion 6 months after surgery	FACT‐B HADS
(Brown et al. 2012), USA	17/15	/	Total knee arthroplasty surgery	Participants designated to the prehabilitation group were directed to engage in three 45‐min sessions of prehabilitation weekly before their operation. A session of prehabilitation of a warm‐up, 10 strength exercises, six stretching exercises, three step exercises, and a cool‐down	Participants in the control group got standard treatment before and after their operation	3 months after surgery	SF–36
(Coca‐Martinez et al. 2023), Canada	13/14	71.7 ± 7.4 69.5 ± 8.1	Endovascular revascularization	Multimodal prehabilitation (MP) included one weekly supervised exercise session, a home‐based exercise regimen, food counseling and supplements, smoking cessation interventions, and psychological support	Participants in the control group got regular care at our hospital, which included walking instruction based on the most recent guidelines from the Society of Vascular Surgery (SVS)	Baseline 12 weeks after baseline assessment 3 months after finishing the program 6 months after finishing the program 12 months after finishing the program	SF–36 HADS
(Ferrara et al. 2008), Italy	11/12	63.82 ± 9.01 63.08 ± 6.89	Hip arthroplasty	The research group participated in an educational and physiotherapy program 1 month prior to surgery	The control group engaged in exercise just post‐surgery	1 month before surgery The day before surgery up to 15 days 4 weeks post surgery 3 months post surgery	SF‐36 Mental composite score (MCS)
(Furze et al. 2009), UK	100/104	64.25 ± 8.81 65.29 ± 8.51	Coronary artery bypass graft surgery	The HeartOp Program helps patients manage heart health through a booklet addressing cardiac myths, risk reduction, and recovery expectations. It includes a relaxation audio and a diary for tracking goals. A facilitator guides the patient in overcoming misconceptions, setting and tracking goals, and provides follow‐up support via phone to adjust goals as needed	The intervention involving nurse education and counseling	Baseline (all data) After the 3rd phone call of the intervention 6 weeks after surgery 3 months after surgery 6 months after surgery	EQ‐5D State scale of the State Trait Anxiety Inventory [STAI] Cardiac Depression Scale [CDS]
(Heiman et al. 2022), Sweden	139/148	/	Breast cancer surgery	Patients in the intervention group were instructed by a physiotherapist to engage in 30 min of moderate‐intensity aerobic exercise daily, commencing 2 ± 1 weeks before to and continuing for 4 weeks following breast cancer surgery. The exercise was conducted autonomously, accompanied by two further calls. Adherence was defined as documenting exercise for over 10 days prior to and 20 days subsequent to surgery	The control group was given concise information on the study's objective—assessing whether physical activity before to and following surgery enhances outcomes—to facilitate informed consent	Baseline 4 weeks after surgery 12 months after surgery	FACT‐B

### 3.2 Risk of Bias

**FIGURE 2:**
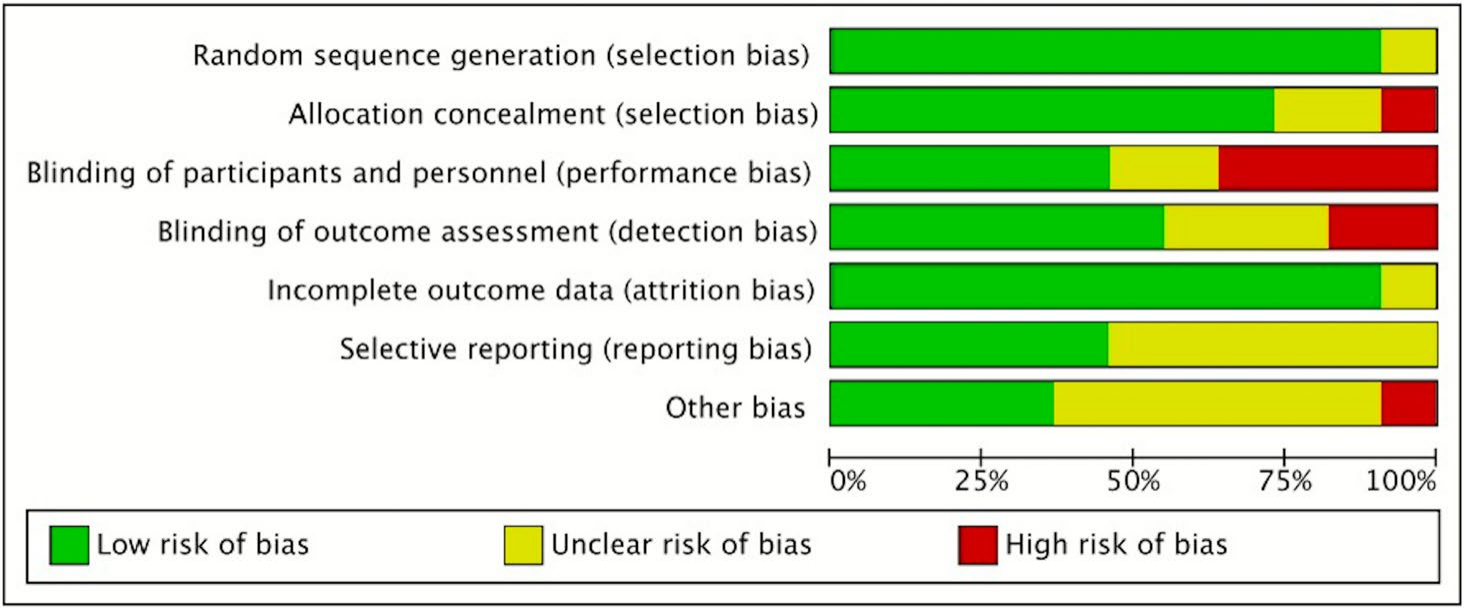
Summary of risk of bias: The review authors' assessments of each methodological quality item are represented as percentages across all included studies. Yellow signifies an ambiguous risk of bias, while red indicates a high risk of bias. Green indicates a minimal risk of bias.

**FIGURE 3:**
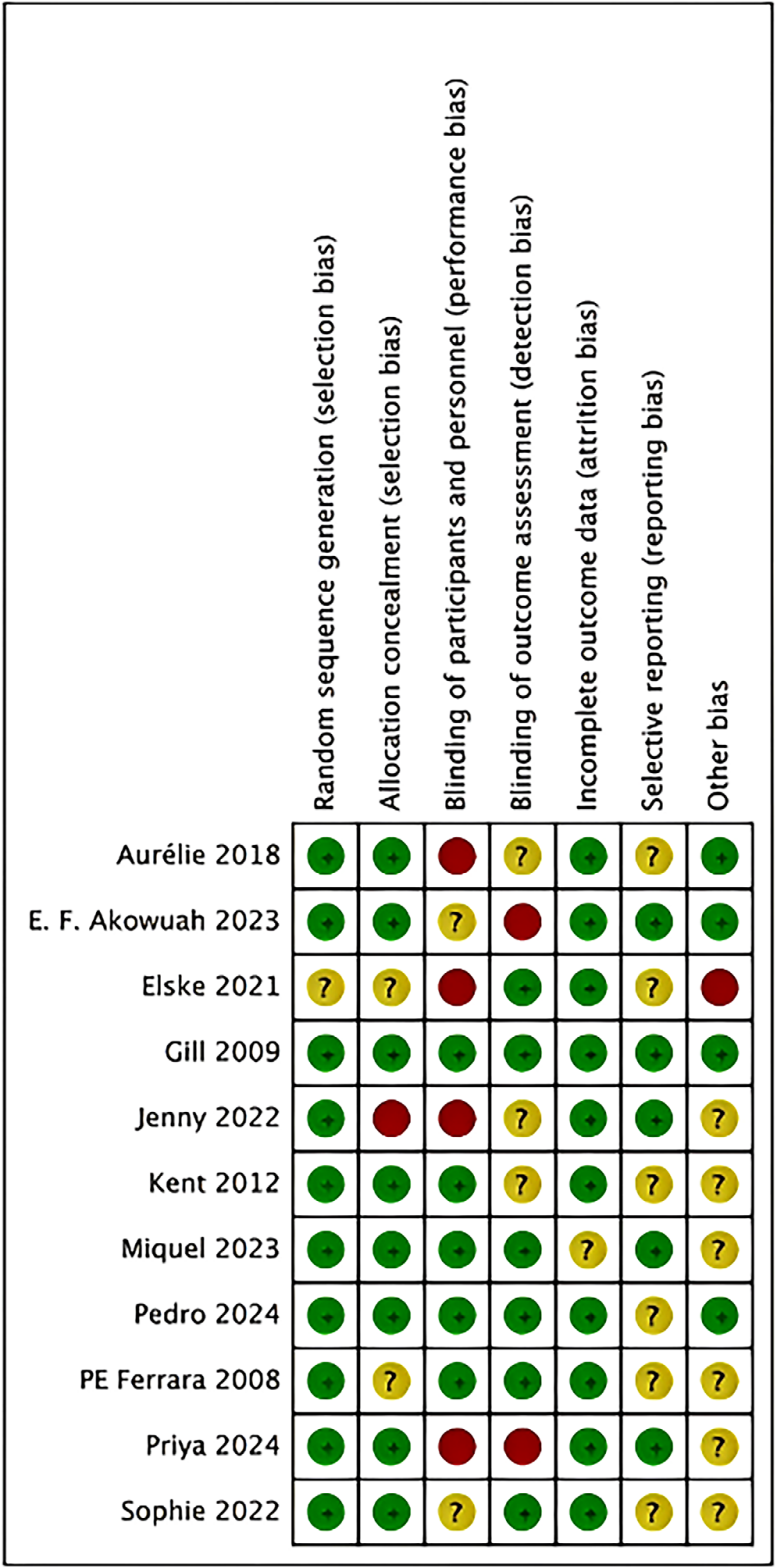
Graph depicting the risk of bias with the Cochrane risk of bias instrument. Red signifies a high danger of prejudice, yellow denotes an ambiguous risk of bias, and green represents a low risk of bias.

Details of the risk of bias assessment are shown in Figures 2 and 3. In general, the study quality assessment reveals mixed results across various domains, with the majority exhibiting a minimal risk of selection and attrition bias. However, significant concerns emerge regarding allocation concealment, with several studies exhibiting uncertain or high risks, thereby raising potential issues of selection bias. Similarly, the performance bias, as well as reporting bias, showed inconsistent risk levels, potentially affecting outcome validity. Selective reporting and other sources of bias also posed varying degrees of uncertainty. Overall, while many studies adhere to rigorous standards, the identified risks in allocation concealment, blinding, and reporting raise critical considerations for interpreting the findings and their implications for future research.

### 3.3 Study Outcomes

A range of outcome measures was delivered, and participants were evaluated at several time points and throughout varied follow‐up durations. Five instruments were employed across four studies to evaluate participants' QOL: the European Organization for Research and Treatment of Cancer (EORTC) Quality of Life Questionnaire C30 (QLQ‐C30) (Allen et al. 2022; Machado et al. 2024), the Standard Form‐36 Health Survey (SF–36) (Berkvens et al. 2021; Brown et al. 2012; Coca‐Martinez et al. 2023; Ferrara et al. 2008), the Functional Assessment of Cancer Therapy‐Breast (FACT‐B) (Brahmbhatt et al. 2024; Heiman et al. 2022), the weight‐related quality of life (Baillot et al. 2018), and the EQ‐5D (Furze et al. 2009). Two scales were used in four studies to measure the effectiveness of the intervention on participants' anxiety. The Hospital Anxiety and Depression Scale (HADS) was used in most studies (Akowuah et al. 2023; Brahmbhatt et al. 2024; Coca‐Martinez et al. 2023), and the other measurements included Becks' Anxiety Inventory (BAI) (Allen et al. 2022). Participants' depression was captured in five studies using the Becks' Depression Inventory II (BDI‐II) (Allen et al. 2022), the Hospital Anxiety and Depression Scale (HADS) (Akowuah et al. 2023; Brahmbhatt et al. 2024; Coca‐Martinez et al. 2023), and the Cardiac Depression Scale (CDS) (Furze et al. 2009).

#### 3.3.1 Effect of Prehabilitation on QoL

**FIGURE 4:**
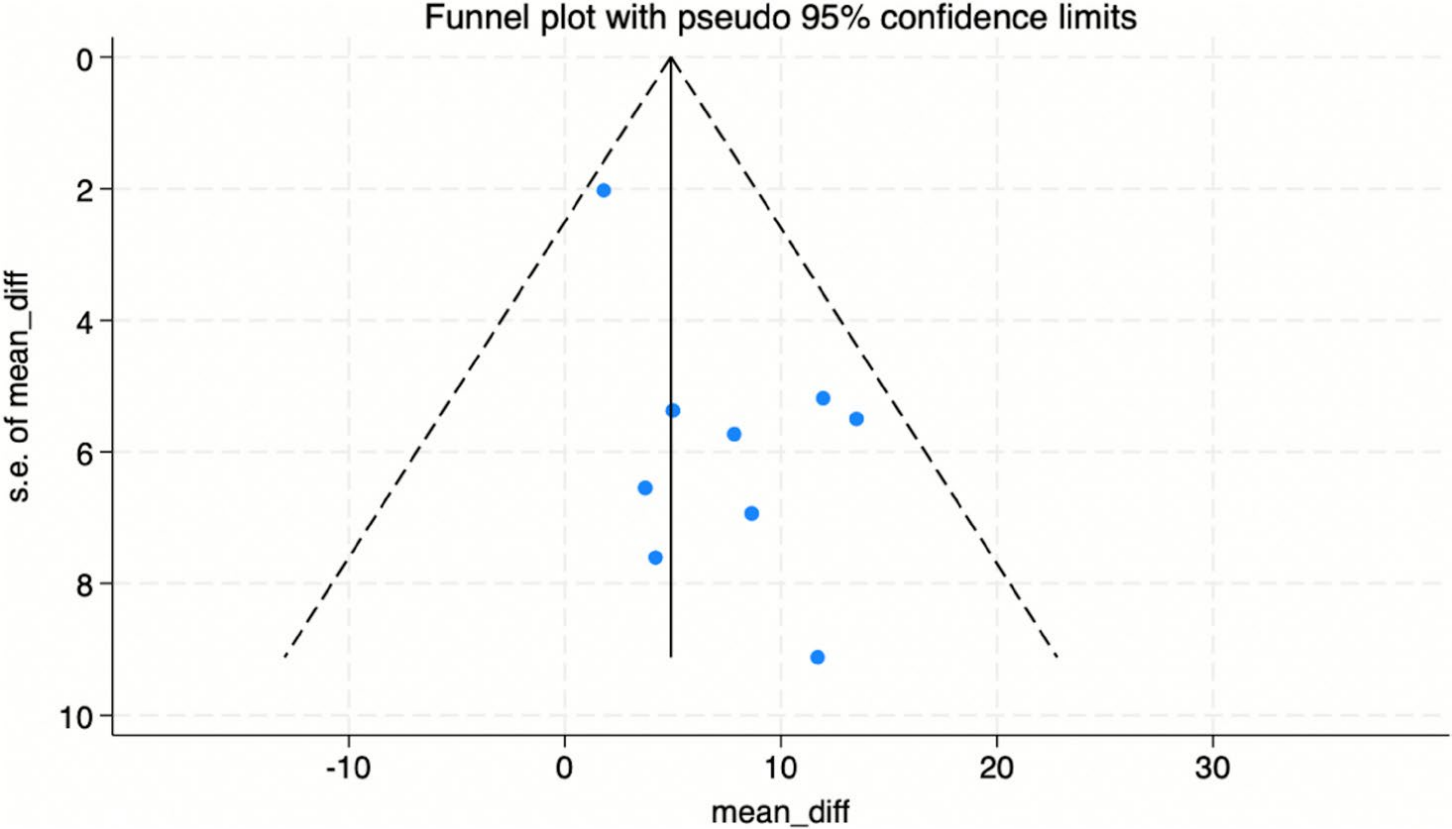
Effect of prehabilitation on QoL: funnel plot.

Ten out of eleven papers documented the effect of prehabilitation on the QoL of postoperative patients, involving 363 patients in the intervention groups and 377 patients in the matched control groups. The overall effect size was estimated to be 4.68 (95% CI: 1.18, 8.18), indicating an overall positive impact of prehabilitation on patients' QoL (*Z* = 2.62, *p* = 0.009). Heterogeneity among the studies was moderate, with a Chi‐squared value of 18.97 (df = 9, *p* = 0.03) and an *I* ^*2*^ statistic of 53%. Meanwhile, it was found that the overall heterogeneity decreased from the original *I* ^*2*^ = 53% to *I* ^*2*^ = 0% after excluding the Akowuah et al. (2023) trial, indicating it may be the origin of the heterogeneity in prehabilitation. When this study was removed, the overall CI widened, leading to the loss of statistical significance. Funnel plot (Figure 4) and Egger's test (*p* = 0.028) indicated there was significant risk of publication bias for prehabilitation.

**FIGURE 5:**
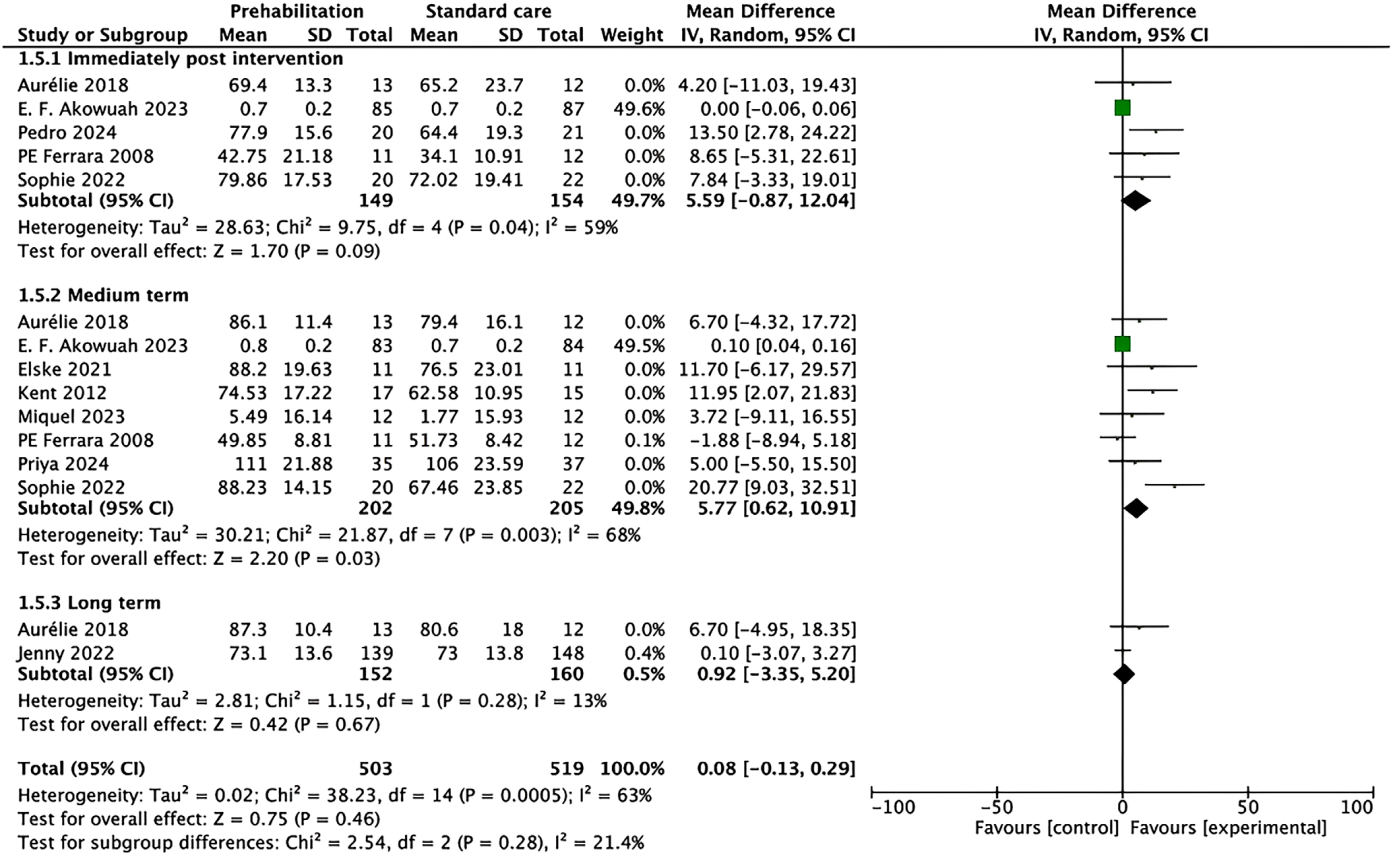
Subgroup analysis of the duration of follow‐up duration using the random‐effect model.

The subgroup analysis based on follow‐up duration revealed a potential beneficial effect of prehabilitation on QoL primarily in the medium term, with an overall effect size of 5.77 (95% CI: [0.62, 10.91], *p* = 0.03). However, significant heterogeneity was noted in both short‐term (*I* ^2^ = 59%, *p* = 0.04) and medium‐term (*I* ^2^ = 68%, *p* = 0.003) groups, indicating variability among the studies. In contrast, no significant impact was observed in the long‐term follow‐up (effect size: 0.92, 95% CI: [−3.35, 5.20], *p* = 0.67) with low heterogeneity (*I* ^2^ = 13%, *p* = 0.67) (Figure 5). Subgroup analysis by surgical type suggested potential differences in the magnitude of QoL improvement. While most subgroups showed positive trends, the small number of studies within each surgical category limited definitive conclusions regarding the influence of surgical type on prehabilitation effectiveness.

#### 3.3.2 Effect of Prehabilitation on Anxiety

**FIGURE 6:**
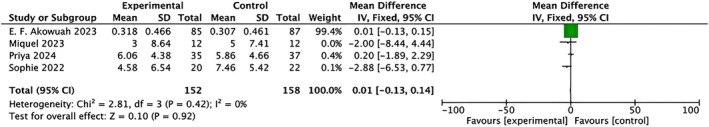
Effectiveness of prehabilitation on anxiety.

Data from four studies were utilized to analyze the effectiveness of prehabilitation on patients' anxiety. Given the minimal heterogeneity (*I* ^*2*^ = 0%, *p* = 0.42) across these studies, a fixed effects model was adopted. The meta‐analysis indicated that prehabilitation had no significant effect on reducing patients' anxiety (SMD = 0.01, 95% CI [−0.13–0.14], *p* = 0.92) (Figure 6). The sensitivity analysis indicated that the result was robust.

#### 3.3.3 Effect of Prehabilitation on Depression

**FIGURE 7:**
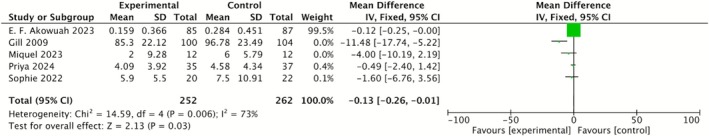
Effectiveness of prehabilitation on depression.

Five studies (Akowuah et al. 2023; Allen et al. 2022; Brahmbhatt et al. 2024; Coca‐Martinez et al. 2023; Furze et al. 2009) contributed data on depression, encompassing 514 people, with 252 in the intervention groups and 262 in the control groups. The aggregated findings indicated a considerable effect size, with a statistically significant reduction in depression following the intervention (SMD = −0.13, 95% CI: −0.26 to −0.01, *Z* = 2.13, *p* = 0.03). Statistical heterogeneity was seen among the studies (*I* ^2^ = 73%, *p* < 0.01), although sensitivity analysis indicated that no one trial might have affected the outcome (Figure 7).

## 4 Discussion

The results of this meta‐analysis indicate a significant positive impact of prehabilitation on the QoL of postoperative patients, as evidenced by a large effect size. This finding aligns with some earlier research that suggested prehabilitation could enhance postoperative outcomes and recovery (Lambert et al. 2021; Punnoose et al. 2023; Skořepa et al. 2024). However, it diverges from others that reported minimal to no improvement in QoL (Seth et al. 2024; Tukanova et al. 2022; Zhang et al. 2024). The variation in outcomes may be attributable to differences in study design, patient populations, types of surgical procedures, and the specific interventions employed during the prehabilitation phase.

One potential explanation for the observed positive effect on QoL is the multifaceted nature of prehabilitation, which typically includes physical exercise, nutritional optimization, and psychological support (Ditmyer et al. 2002). This comprehensive approach may better prepare patients for surgery, both physically and emotionally, thereby facilitating a smoother recovery process. Previous studies have highlighted that physical conditioning prior to surgery can improve functional capacity (Awasthi et al. 2019; Gillis et al. 2014; Minnella et al. 2017), reduce complications (Berkel et al. 2022; Souwer et al. 2018), and the length of hospital stays (Chia et al. 2016; Souwer et al. 2018), ultimately leading to an improved QoL postoperatively. Furthermore, the psychological aspects of prehabilitation, such as enhanced coping strategies and reduced postoperative stress, may directly contribute to improved QoL (Aglio et al. 2022), although our findings on anxiety suggest that its effect in this domain may be limited.

Subgroup analysis by surgical type, while limited by the number of studies per category, suggests that the effect of prehabilitation on QoL might vary across different surgical contexts. For instance, the physiological and psychological demands, as well as recovery trajectories, differ significantly between cardiac, oncologic, and orthopedic surgeries. The mechanisms underlying the benefits of prehabilitation may thus be surgery‐specific. In major surgeries like cardiac or cancer resections, where physiological reserve is crucial, prehabilitation's physical component might be paramount. In surgeries where functional recovery and pain are primary concerns (e.g., arthroplasty), a combination of physical and psychological interventions might be more impactful. Future research with larger samples within specific surgical groups is needed to elucidate these mechanisms and optimize prehabilitation protocols accordingly.

The subgroup analysis revealed that the beneficial effects of prehabilitation were particularly pronounced in the medium term. This could suggest that the interactions between prehabilitation and recovery outcomes may become more evident after the initial postoperative period. The heterogeneity observed in the medium‐term follow‐up (*I* ^2^ = 68%) could be due to differences in the prehabilitation programs' intensity, duration, and components across studies. In contrast, the lack of significant impact in the long‐term follow‐up is intriguing. This could be due to the waning effects of prehabilitation as patients return to their preoperative lifestyle habits or face new postoperative challenges. Additionally, long‐term outcomes might be influenced by factors beyond the scope of prehabilitation, such as the quality of postoperative care and the patient's home environment.

The findings on anxiety levels present a more complex picture. Our meta‐analysis showed no significant impact of prehabilitation on reducing anxiety, which diverges from some studies that reported positive outcomes in this area (Elsherbini and Carli 2022; Minnella et al. 2017). The low heterogeneity (*I* ^2^ = 0%) among studies in this analysis suggests that the interventions were fairly consistent in their design and implementation. However, the overall non‐significant effect on anxiety may be due to the varying effectiveness of psychological interventions across different patient populations. Furthermore, the limited effect of prehabilitation on anxiety could imply that while physical preparedness is enhanced, the psychological benefits may not be as pronounced or may require additional interventions such as targeted counseling or support groups (Taha et al. 2021). It is possible that prehabilitation programs need to be more tailored to individual psychological needs to effectively reduce anxiety. Future research should explore personalized prehabilitation approaches that consider patients' baseli ne anxiety levels and psychological profiles.

In terms of depression, our analysis underscores a substantial improvement associated with prehabilitation, which is consistent with the notion that enhancing physical health can have profound impacts on mental well‐being (Ashdown‐Franks et al. 2020; Milaneschi et al. 2019; Rebar et al. 2015). The significant heterogeneity observed in these studies indicates that various factors, such as the type of interventions, patient demographics, and specific surgical contexts, must be considered when interpreting these results. The persistent effect of prehabilitation on reducing depression can be attributed to the combined physical and psychological benefits that prehabilitation aims to provide, including community support and social engagement which were not always addressed sufficiently in previous studies.

Overall, while our findings support the efficacy of prehabilitation in improving QoL and reducing depression among postoperative patients, they also highlight the need for more standardized interventions and assessment measures in future research. As prehabilitation continues to evolve, understanding patient‐specific factors and tailoring approaches to individual needs could enhance its effectiveness across different surgical populations. Future studies should also explore the long‐term effects of prehabilitation, particularly regarding sustained improvement in QoL and psychological health, to better inform clinical practice and optimize patient care throughout the surgical journey.

4.1

While the current meta‐analysis provides valuable insights into the effects of prehabilitation on quality of life, anxiety, and depression in postoperative patients, there are several limitations that should be considered: (1) The moderate to high heterogeneity observed in the QoL and depression analyses indicates variability in study designs, populations, and interventions, which may affect the generalizability of findings. (2) The assessment of anxiety and depression was based on a limited number of studies, which may have affected the power of the meta‐analysis to detect statistically significant effects. (3) There might be publication bias, as shown by the asymmetric funnel plot and Egger's test result. Although we searched for unpublished studies in grey literature and registries, we found none that met our criteria. Therefore, this review only includes published data, potentially missing other relevant studies. (4) The preoperative rehabilitation programs included in the studies varied significantly in specific contents (e.g., form and intensity of exercise, depth of psychological interventions), implementers (e.g., physiotherapists, nurses, psychologists), and intervention durations. This clinical and methodological heterogeneity is an important factor potentially affecting the overall effectiveness and observed heterogeneity, and limits the ability to draw definitive conclusions about the optimal prehabilitation protocol. Future research efforts should aim to address these limitations by incorporating standardized methodologies, larger sample sizes, and comprehensive evaluations of prehabilitation programs.

## 5 Conclusions

This meta‐analysis highlights the positive impact of prehabilitation on improving postoperative patients' quality of life and reducing depression, while noting negligible effects on anxiety. The overall effect size for QoL was significant, particularly in the medium term, although the benefits did not extend into the long term. High heterogeneity among studies indicates variability that warrants cautious interpretation. These findings suggest that prehabilitation can be a valuable component of postoperative care for enhancing QoL and addressing depression, but further research is needed to understand how these benefits can be maintained and to evaluate the effects on anxiety more comprehensively.

## Funding

This work was supported by the 2024 Nursing Research Fund (General Project) of Tongji Hospital, Tongji Medical College, Huazhong University of Science and Technology, 2024D50.

## Ethics Statement

The study was conducted in strict compliance with ethical principles. All procedures involving human participants adhered to the ethical standards established by the relevant institutional and national research ethics committees, as well as the tenets of the Declaration of Helsinki (1964) and its subsequent revisions.

## Consent

Informed consent was obtained from all individual participants included in the study.

## Conflicts of Interest

The authors declare no conflicts of interest.

## Data Availability

The data sets used and/or analyzed in this study can be obtained from the corresponding author upon request.

## References

[pchj70080-bib-0001] Aglio, L. S. , E. Mezzalira , L. Mendez‐Pino , et al. 2022. “Surgical Prehabilitation: Strategies and Psychological Intervention to Reduce Postoperative Pain and Opioid Use.” Anesthesia & Analgesia 134, no. 5: 1106–1111. 10.1213/ANE.0000000000005963. 35427271

[pchj70080-bib-0002] Akowuah, E. F. , J. M. Wagnild , M. Bardgett , et al. 2023. “A Randomised Controlled Trial of Prehabilitation in Patients Undergoing Elective Cardiac Surgery.” Anaesthesia 78, no. 9: 1120–1128. 10.1111/anae.16072. 37402352

[pchj70080-bib-0003] Allen, S. K. , V. Brown , D. White , et al. 2022. “Multimodal Prehabilitation During Neoadjuvant Therapy Prior to Esophagogastric Cancer Resection: Effect on Cardiopulmonary Exercise Test Performance, Muscle Mass and Quality of Life—A Pilot Randomized Clinical Trial.” Annals of Surgical Oncology 29, no. 3: 1839–1850. 10.1245/s10434-021-11002-0. 34725764

[pchj70080-bib-0004] Ashdown‐Franks, G. , J. Firth , R. Carney , et al. 2020. “Exercise as Medicine for Mental and Substance Use Disorders: A Meta‐Review of the Benefits for Neuropsychiatric and Cognitive Outcomes.” Sports Medicine 50, no. 1: 151–170. 10.1007/s40279-019-01187-6. 31541410

[pchj70080-bib-0005] Awasthi, R. , E. M. Minnella , V. Ferreira , A. V. Ramanakumar , C. Scheede‐Bergdahl , and F. Carli . 2019. “Supervised Exercise Training With Multimodal Pre‐Habilitation Leads to Earlier Functional Recovery Following Colorectal Cancer Resection.” Acta Anaesthesiologica Scandinavica 63, no. 4: 461–467. 10.1111/aas.13292. 30411316

[pchj70080-bib-0006] Baillot, A. , C.‐A. Vallée , W. M. Mampuya , et al. 2018. “Effects of a Pre‐Surgery Supervised Exercise Training 1 Year After Bariatric Surgery: A Randomized Controlled Study.” Obesity Surgery 28, no. 4: 955–962. 10.1007/s11695-017-2943-8. 28963710

[pchj70080-bib-0007] Berkel, A. E. M. , B. C. Bongers , H. Kotte , et al. 2022. “Effects of Community‐Based Exercise Prehabilitation for Patients Scheduled for Colorectal Surgery With High Risk for Postoperative Complications: Results of a Randomized Clinical Trial.” Annals of Surgery 275, no. 2: e299–e306. 10.1097/SLA.0000000000004702. 33443905 PMC8746915

[pchj70080-bib-0008] Berkvens, E. H. M. , J. A. Wegdam , R. J. A. Visser , N. D. Bouvy , S. W. Nienhuijs , and T. S. De Vries Reilingh . 2021. “Preoperative Exercise Therapy Preventing Postoperative Complications Following Complex Abdominal Wall Reconstruction: A Feasibility Study.” International Journal of Abdominal Wall and Hernia Surgery 4, no. 3: 103–108. 10.4103/ijawhs.ijawhs_33_21.

[pchj70080-bib-0009] Brahmbhatt, P. , N. J. Look Hong , A. Sriskandarajah , et al. 2024. “A Feasibility Randomized Controlled Trial of Prehabilitation During Neoadjuvant Chemotherapy for Women With Breast Cancer: A Mixed Methods Study.” Annals of Surgical Oncology 31, no. 4: 2261–2271. 10.1245/s10434-023-14851-z. 38219003

[pchj70080-bib-0010] Brown, K. , J. A. Brosky , R. Topp , and A. S. Lajoie . 2012. “Prehabilitation and Quality of Life Three Months After Total Knee Arthroplasty: A Pilot Study.” Perceptual and Motor Skills 115, no. 3: 765–774. 10.2466/15.06.10.PMS.115.6.765-774. 23409591

[pchj70080-bib-0011] Chia, C. L. K. , S. K. Mantoo , and K. Y. Tan . 2016. “‘Start to Finish Trans‐Institutional Transdisciplinary Care’: A Novel Approach Improves Colorectal Surgical Results in Frail Elderly Patients.” Colorectal Disease 18, no. 1: 43–50. 10.1111/codi.13166. 26500155

[pchj70080-bib-0012] Coca‐Martinez, M. , E. Girsowicz , R. J. Doonan , et al. 2023. “Multimodal Prehabilitation for Peripheral Arterial Disease Patients With Intermittent Claudication‐A Pilot Randomized Controlled Trial.” Annals of Vascular Surgery S0890, no. 23: 007677. 10.1016/j.avsg.2023.09.101. 37949167

[pchj70080-bib-0013] Deprato, A. , K. Verhoeff , K. Purich , J. Y. Kung , D. L. Bigam , and K. Z. Dajani . 2022. “Surgical Outcomes and Quality of Life Following Exercise‐Based Prehabilitation for Hepato‐Pancreatico‐Biliary Surgery: A Systematic Review and Meta‐Analysis.” Hepatobiliary & Pancreatic Diseases International 21, no. 3: 207–217. 10.1016/j.hbpd.2022.02.004. 35232658

[pchj70080-bib-0014] Ditmyer, M. M. , R. Topp , and M. Pifer . 2002. “Prehabilitation in Preparation for Orthopaedic Surgery.” Orthopaedic Nursing 21, no. 5: 43–54. 10.1097/00006416-200209000-00008. 12432699

[pchj70080-bib-0015] Elsherbini, N. , and F. Carli . 2022. “Advocating for Prehabilitation for Patients Undergoing Gynecology‐Oncology Surgery.” European Journal of Surgical Oncology 48, no. 9: 1875–1881. 10.1016/j.ejso.2022.04.021. 35534307

[pchj70080-bib-0016] Ferrara, P. , A. Rabini , L. Maggi , et al. 2008. “Effect of Pre‐Operative Physiotherapy in Patients With End‐Stage Osteoarthritis Undergoing Hip Arthroplasty.” Clinical Rehabilitation 22: 977–986. 10.1177/0269215508094714. 18955429

[pchj70080-bib-0017] Furze, G. , J. C. Dumville , J. N. V. Miles , K. Irvine , D. R. Thompson , and R. J. P. Lewin . 2009. “Prehabilitation Prior to CABG Surgery Improves Physical Functioning and Depression.” International Journal of Cardiology 132, no. 1: 51–58. 10.1016/j.ijcard.2008.06.001. 18703241 PMC2643012

[pchj70080-bib-0018] Gillis, C. , K. Buhler , L. Bresee , et al. 2018. “Effects of Nutritional Prehabilitation, With and Without Exercise, on Outcomes of Patients Who Undergo Colorectal Surgery: A Systematic Review and Meta‐Analysis.” Gastroenterology 155, no. 2: 391–410.e4. 10.1053/j.gastro.2018.05.012. 29750973

[pchj70080-bib-0019] Gillis, C. , C. Li , L. Lee , et al. 2014. “Prehabilitation Versus Rehabilitation: A Randomized Control Trial in Patients Undergoing Colorectal Resection for Cancer.” Anesthesiology 121, no. 5: 937–947. 10.1097/ALN.0000000000000393. 25076007

[pchj70080-bib-0020] Han, C. , H. Ji , Y. Guo , et al. 2023. “Effect of Subanesthetic Dose of Esketamine on Perioperative Neurocognitive Disorders in Elderly Undergoing Gastrointestinal Surgery: A Randomized Controlled Trial.” Drug Design, Development and Therapy 17: 863–873. 10.2147/DDDT.S401161. 36974331 PMC10039635

[pchj70080-bib-0021] Heiman, J. , A. Onerup , D. Bock , E. Haglind , and R. Olofsson Bagge . 2022. “The Effect of Nonsupervised Physical Activity Before and After Breast Cancer Surgery on Quality of Life: Results From a Randomized Controlled Trial (PhysSURG‐B).” Scandinavian Journal of Surgery 111, no. 4: 75–82. 10.1177/14574969221123389. 36113110

[pchj70080-bib-0022] Kenny, E. , H. Samavat , R. Touger‐Decker , J. S. Parrott , L. Byham‐Gray , and D. A. August . 2022. “Adverse Perioperative Outcomes Among Patients Undergoing Gastrointestinal Cancer Surgery: Quantifying Attributable Risk From Malnutrition.” Journal of Parenteral and Enteral Nutrition 46, no. 3: 517–525. 10.1002/jpen.2200. 34057749

[pchj70080-bib-0023] Lambert, J. E. , L. D. Hayes , T. J. Keegan , D. A. Subar , and C. J. Gaffney . 2021. “The Impact of Prehabilitation on Patient Outcomes in Hepatobiliary, Colorectal, and Upper Gastrointestinal Cancer Surgery: A PRISMA‐Accordant Meta‐Analysis.” Annals of Surgery 274, no. 1: 70–77. 10.1097/SLA.0000000000004527. 33201129

[pchj70080-bib-0024] Li, C. , F. Carli , L. Lee , et al. 2013. “Impact of a Trimodal Prehabilitation Program on Functional Recovery After Colorectal Cancer Surgery: A Pilot Study.” Surgical Endoscopy 27, no. 4: 1072–1082. 10.1007/s00464-012-2560-5. 23052535

[pchj70080-bib-0025] Liang, J. , L. Wang , J. Song , et al. 2024. “The Impact of Nursing Interventions on the Rehabilitation Outcome of Patients After Lumbar Spine Surgery.” BMC Musculoskeletal Disorders 25, no. 1: 354. 10.1186/s12891-024-07419-9. 38704573 PMC11069211

[pchj70080-bib-0026] Lotzke, H. , H. Brisby , A. Gutke , et al. 2019. “A Person‐Centered Prehabilitation Program Based on Cognitive‐Behavioral Physical Therapy for Patients Scheduled for Lumbar Fusion Surgery: A Randomized Controlled Trial.” Physical Therapy 99, no. 8: 1069–1088. 10.1093/ptj/pzz020. 30951604 PMC6665875

[pchj70080-bib-0027] Machado, P. , S. Pimenta , A. L. Garcia , et al. 2024. “Effect of Preoperative Home‐Based Exercise Training on Quality of Life After Lung Cancer Surgery: A Multicenter Randomized Controlled Trial.” Annals of Surgical Oncology 31, no. 2: 847–859. 10.1245/s10434-023-14503-2. 37934383 PMC10761542

[pchj70080-bib-0028] Madsen, H. J. , W. G. Henderson , A. R. Dyas , et al. 2023. “Inpatient Versus Outpatient Surgery: A Comparison of Postoperative Mortality and Morbidity in Elective Operations.” World Journal of Surgery 47, no. 3: 627–639. 10.1007/s00268-022-06819-z. 36380104

[pchj70080-bib-0029] Milaneschi, Y. , W. K. Simmons , E. F. C. Van Rossum , and B. W. Penninx . 2019. “Depression and Obesity: Evidence of Shared Biological Mechanisms.” Molecular Psychiatry 24, no. 1: 18–33. 10.1038/s41380-018-0017-5. 29453413

[pchj70080-bib-0030] Minnella, E. M. , G. Bousquet‐Dion , R. Awasthi , C. Scheede‐Bergdahl , and F. Carli . 2017. “Multimodal Prehabilitation Improves Functional Capacity Before and After Colorectal Surgery for Cancer: A Five‐Year Research Experie nce.” Acta Oncologica 56, no. 2: 295–300. 10.1080/0284186X.2016.1268268. 28079430

[pchj70080-bib-0031] Page, M. J. , J. E. McKenzie , P. M. Bossuyt , et al. 2021. “The PRISMA 2020 Statement: An Updated Guideline for Reporting Systematic Reviews.” British Medical Journal 372: n71. 10.1136/bmj.n71. 33782057 PMC8005924

[pchj70080-bib-0032] Peng, Y.‐N. , L. Jin , E.‐J. Peng , and L. Zhang . 2023. “Perioperative Care Based on Roy Adaptation Model in Elderly Patients With Benign Prostatic Hyperplasia: Impact on Psychological Well‐Being, Pain, and Quality of Life.” BMC Urology 23, no. 1: 172. 10.1186/s12894-023-01343-1. 37891515 PMC10612228

[pchj70080-bib-0033] Punnoose, A. , L. S. Claydon‐Mueller , O. Weiss , J. Zhang , A. Rushton , and V. Khanduja . 2023. “Prehabilitation for Patients Undergoing Orthopedic Surgery: A Systematic Review and Meta‐Analysis.” JAMA Network Open 6, no. 4: e238050. 10.1001/jamanetworkopen.2023.8050. 37052919 PMC10102876

[pchj70080-bib-0034] Rebar, A. L. , R. Stanton , D. Geard , C. Short , M. J. Duncan , and C. Vandelanotte . 2015. “A Meta‐Meta‐Analysis of the Effect of Physical Activity on Depression and Anxiety in Non‐Clinical Adult Populations.” Health Psychology Review 9, no. 3: 366–378. 10.1080/17437199.2015.1022901. 25739893

[pchj70080-bib-0035] Seth, I. , G. Bulloch , K. R. Qin , et al. 2024. “Pre‐Rehabilitation Interventions for Patients With Head and Neck Cancers: A Systematic Review and Meta‐Analysis.” Head & Neck 46, no. 1: 86–117. 10.1002/hed.27561. 37897197

[pchj70080-bib-0036] Skořepa, P. , K. L. Ford , A. Alsuwaylihi , et al. 2024. “The Impact of Prehabilitation on Outcomes in Frail and High‐Risk Patients Undergoing Major Abdominal Surgery: A Systematic Review and Meta‐Analysis.” Clinical Nutrition 43, no. 3: 629–648. 10.1016/j.clnu.2024.01.020. 38306891

[pchj70080-bib-0037] Souwer, E. T. D. , E. Bastiaannet , S. De Bruijn , et al. 2018. “Comprehensive Multidisciplinary Care Program for Elderly Colorectal Cancer Patients: From Prehabilitation to Independence.” European Journal of Surgical Oncology 44, no. 12: 1894–1900. 10.1016/j.ejso.2018.08.028. 30266205

[pchj70080-bib-0038] Steffens, D. , P. R. Beckenkamp , M. Hancock , M. Solomon , and J. Young . 2018. “Preoperative Exercise Halves the Postoperative Complication Rate in Patients With Lung Cancer: A Systematic Review of the Effect of Exercise on Complications, Length of Stay and Quality of Life in Patients With Cancer.” British Journal of Sports Medicine 52, no. 5: 344. 10.1136/bjsports-2017-098032. 29437041

[pchj70080-bib-0039] Taha, A. , S. Taha‐Mehlitz , V. E. Staartjes , et al. 2021. “Association of a Prehabilitation Program With Anxiety and Depression Before Colorectal Surgery: A Post Hoc Analysis of the pERACS Randomized Controlled Trial.” Langenbeck's Archives of Surgery 406, no. 5: 1553–1561. 10.1007/s00423-021-02158-0. 33782738

[pchj70080-bib-0040] Timmerman, H. , J. F. De Groot , H. J. Hulzebos , R. De Knikker , H. E. M. Kerkkamp , and N. L. U. Van Meeteren . 2011. “Feasibility and Preliminary Effectiveness of Preoperative Therapeutic Exercise in Patients With Cancer: A Pragmatic Study.” Physiotherapy Theory and Practice 27, no. 2: 117–124. 10.3109/09593981003761509. 20690877

[pchj70080-bib-0041] Tukanova, K. H. , S. Chidambaram , N. Guidozzi , G. B. Hanna , A. H. McGregor , and S. R. Markar . 2022. “Physiotherapy Regimens in Esophagectomy and Gastrectomy: A Systematic Review and Meta‐Analysis.” Annals of Surgical Oncology 29, no. 5: 3148–3167. 10.1245/s10434-021-11122-7. 34961901 PMC8990957

[pchj70080-bib-0042] Zhang, J. , Y. Hu , H. Deng , Z. Huang , J. Huang , and Q. Shen . 2024. “Effect of Preoperative Lifestyle Management and Prehabilitation on Postoperative Capability of Colorectal Cancer Patients: A Systematic Review and Meta‐Analysis.” Integrative Cancer Therapies 23: 15347354241235590. 10.1177/15347354241235590. 38439687 PMC10916464

